# PediHaplotyper: software for consistent assignment of marker haplotypes in pedigrees

**DOI:** 10.1007/s11032-016-0539-y

**Published:** 2016-08-08

**Authors:** Roeland E. Voorrips, Marco C. A. M. Bink, Johannes W. Kruisselbrink, Herma J. J. Koehorst-van Putten, W. Eric van de Weg

**Affiliations:** 1 Plant Breeding, Wageningen University and Research Centre, P.O. Box 386, 6700 AJ Wageningen, The Netherlands; 2 Biometris, Wageningen University and Research Centre, P.O. Box 16, 6700 AA Wageningen, The Netherlands; 3 Hendrix Genetics Research, P.O. Box 114, 5830 AC Boxmeer, The Netherlands

**Keywords:** SNP, Haplotyping, Haploblock, Pedigree

## Abstract

In the study of large outbred pedigrees with many founders, individual bi-allelic markers, such as SNP markers, carry little information. After phasing the marker genotypes, multi-allelic loci consisting of groups of closely linked markers can be identified, which are called “haploblocks”. Here, we describe PediHaplotyper, an R package capable of assigning consistent alleles to such haploblocks, allowing for missing and incorrect SNP data. These haploblock genotypes are much easier to interpret by the human investigator than the original SNP data and also allow more efficient QTL analyses that require less memory and computation time.

**Electronic supplementary material:**

The online version of this article (doi:10.1007/s11032-016-0539-y) contains supplementary material, which is available to authorized users.

## Introduction

The current high-density, affordable SNP genotyping platforms create new opportunities to track the transmission of genomic regions through pedigrees, for instance in crop breeding programs. The genetic data can serve various purposes, such as to verify or correct pedigree relations, to infer unknown pedigree relations, e.g. the identification of a parent from a set of candidates, to infer identity-by-descent (IBD) for genomic regions among individuals and to perform quantitative trait locus (QTL) analyses.

Bi-allelic SNP markers do not carry much information individually, and in situations where multiple functional alleles of genes may be segregating (such as in breeding pedigrees), this means that large numbers of SNPs need to be analysed. While this is possible, during QTL mapping IBD relations may not always be inferred correctly and may therefore not be able to extract all information present in the data. Also, in order to enable genetic analyses in pedigrees, the data should be of high quality; although incorrect SNP genotypes may not be directly apparent, they are likely to result in erroneous phasing and IBD estimates and in inaccuracies in QTL mapping. In diploid bi-parental experimental populations genotyping errors are relatively easy to spot, but in pedigrees with multiple founders, extending over multiple generations and where many individuals have only few offspring, the detection of genotyping errors is more challenging, among others due to the more frequent occurrence of null alleles (Pikunova et al. 2014; Di Guardo et al. 2015).

An alternative to performing genetic analyses directly on high-density SNP data is to add an intermediate step, where several closely linked SNPs are considered to form a single genetic locus, which we call a haploblock. At a haploblock, multiple alleles (SNP haplotypes) can segregate. Potentially, this haploblock approach has several advantages over working with individual SNP genotypes. One advantage is a reduction of the amount of data (and hence also of required computer memory and processing time) without losing information: the number of haploblocks is obviously smaller than the number of individual SNPs. Another advantage is that the haploblock alleles (SNP haplotypes) that occur in the pedigree are known before IBD and QTL analyses are performed, reducing the time spent while dynamically phasing SNP alleles during these analyses. A further, very important advantage is that a smaller number of polymorphic markers are much easier to interpret for the human investigator than a large number of bi-allelic SNPs, for instance in checking consistency of pedigree relations with the marker data and in checking for possible relationships in case of unknown parentages. Here, we present an approach to identify haploblocks and SNP haplotypes and a software package PediHaplotyper that performs some of the steps in this process: the identification of the different haploblock alleles occurring in the pedigree and genotyping of the individuals in terms of haploblock alleles, including error detection and imputation of missing SNP data.

### Approach

The derivation of multi-allelic haploblock genotypes from bi-allelic SNP data in our approach involves three steps: (1) for each SNP marker and each heterozygous individual, determination of which allele was inherited from which (grand)parent (also known as phasing); (2) identification of closely linked groups of SNPs without recombination in most of the pedigree, which we call “haploblocks” (intra-haploblock recombinations cause apparently inconsistent inheritance patterns; therefore, they are only allowed in the final generation, where the recombinant alleles must be replaced by missing data) and (3) assignment of consistent genotypes to all individuals for each haploblock. For step (1) and (2), various software is available, including Beagle (Browning and Browning 2011), AlphaImpute (Hickey et al. 2012) and FlexQTL (Bink et al. 2014). For step (3), we have developed the software package PediHaplotyper. While PediHaplotyper was developed mainly to obtain haploblock genotypes from bi-allelic SNP markers, also multi-allelic markers such as SSRs may be included.

## Implementation

PediHaplotyper is implemented as an R package (R Core Team 2015) and therefore will run on any system for which R is available including the various versions of MS Windows, Linux distributions and Apple iOS. The assignment of marker haplotypes, here called haploblock alleles, is performed with a single function call. The different stages in the assignment are handled by this function and are detailed in the next sections.

PediHaplotyper requires four sources of information as input: the genetic linkage map of the original (SNP) markers, the definition of the haploblocks (i.e. which markers make up each haploblock), the pedigree and the phased marker genotypes for the individuals in the pedigree; optionally additional data such as phenotypes can be supplied as well. Several output files are produced, including (1) the phased multi-allelic haploblock genotypes for QTL mapping, in a generic tab-delimited text file format or as a set of files suitable for import into FlexQTL™, (2) files in Pedimap (Voorrips et al. 2012) format for visualizing the flow of marker alleles and haploblock alleles through the pedigree, including the phenotypic data if these were supplied and (3) diagnostic files. The formats of the input and output files are detailed in the manual (Supplementary file 1).

### Reading of input data

The input data can be read in several formats, including generic tab-delimited text files and a format compatible with FlexQTL™. Checks are performed whether the individuals and markers are consistent between the different files and whether the haploblocks are composed of contiguous markers.

### Assignment of original haploblock alleles

For each haploblock, each sequence of marker alleles that occurs in the pedigree is assigned its own haploblock allele number. These haploblock alleles may contain missing values for some or all markers. In this step, markers that do not exceed a user-defined frequency threshold are rejected. The default of this threshold is a minimum of three occurrences of each of the two (or more) marker alleles.

### Ordering the pedigree

The pedigree is dissected into half-sib (HS) families: each HS family is composed of all individuals that share one parent. This parent may be either the female or the male parent or even both in the case of self-fertilization. Then, these HS families are ordered by decreasing number of individuals, which is the order in which they will be processed in the next stage. Note that, except for the first and last generations, each individual is the parent of one HS family and a member of two (or one) HS families (one for each of its parents, unless only one parent is known).

### Calculation of consistent phased haploblock genotypes

The calculation of the haploblock genotypes is done separately for each haploblock. Following their size-based ordering, all HS families are considered one by one in an iterative process. The ordering of the HS families by size ensures that generally, the families containing most information are used first. If any changes occur during the processing of the whole pedigree (see below), a new iteration is performed. The process has converged once no changes occur any more, or when the same configuration of haploblock genotypes occurs for a second time. The process is stopped when convergence is reached or when a (user specified) maximum number of iterations have passed. Only when convergence has been reached, haploblock genotypes are assigned.

The treatment of an HS family is a rule-based process which starts with grouping the haploblock alleles in the HS family that are inherited from the common parent into groups. Although only one or two alleles can be inherited from the parent, more than two groups may be identified in the HS family due to missing marker data and scoring errors. The grouping is based on compatibility: Two haploblock alleles are compatible if they differ only in the missing marker data and have at least one non-missing marker score in common. The resulting groups are composed of haploblock alleles that are all mutually compatible, and that are incompatible with all alleles in the other groups. For each group, a consensus haplotype is obtained in which for each SNP where at least one of the haploblock alleles has a non-missing value, that value is used. There may also be ungrouped alleles that are compatible with more than one group due to missing marker data; these are in most cases treated as missing values. The grouping is illustrated in Table 1.

**Table 1:**
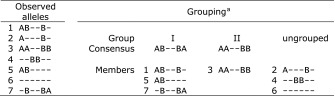
Illustration of the grouping of haploblock alleles comprising a block of six bi-allelic markers

^a^Two groups of haploblock alleles are identified: group I with three observed alleles and group II with one allele. The three ungrouped haploblock alleles are compatible with both groups. A dash indicates an unknown marker allele

Once the alleles of an HS family are grouped, the consensus alleles of these groups are compared to the two parental alleles. Different situations are distinguished based on several criteria, including the number of individuals in the HS family, the number of allele groups identified in the HS family, the number of HS family members in each group, the matching of the parental alleles with each other and with the groups in the HS family. In this step, the aim is to assign consistent haploblock alleles to parent and progeny, i.e. only one or two alleles are inherited from the common parent in the progeny, and these must be identical (including any missing marker data within the haploblock allele) to the two alleles present in the parent. Where necessary, this is achieved by imputing missing marker data or by deleting marker data. For example, in the simplest situation, a HS family has at least 15 members and all their alleles are in one group. Then, it is very likely that the parent is homozygous. Next, the parental alleles are considered in relation to the consensus allele of the progeny. Several situations are possible: (1) One or both alleles of the parent are available and are not in conflict with the progeny group consensus or with each other; in this case, a new consensus is derived by combining these parental alleles with the consensus from the progeny, and this updated consensus allele is imputed for the parent and progeny. (2) The two parental alleles both match the progeny consensus but do not match each other, due to marker(s) in the haploblock for which the parent was genotyped but the progeny was not. In this case, the parent may be heterozygous after all, but it is impossible to assign the parental alleles to the progeny individuals. In assigning consistent alleles to parent and progeny, less information is lost by assigning the progeny consensus to both parental alleles (meaning that the data for the differentiating marker(s) are deleted for the parent) than by assigning missing data for the entire haploblock to the progeny. (3) One or both parental alleles conflict with the group consensus; in that case, the conflicting parental allele(s) is/are rejected (i.e. all marker data for the haploblock are made missing in the parent); if only one parental allele conflicts with the progeny, the other can either match with the progeny consensus allele or be missing and is treated as above under (1). Similar but more complex decision chains are applied when the HS family is smaller than 15 individuals (homozygosity of the parent can then not be inferred from the presence of just one group in the progeny), when not all alleles in the HS family are in the same group or both. These decision chains are documented with comments in the source code. In all cases where missing or conflicting alleles are involved, there are various quantitative considerations that decide which (if any) allele is considered the correct one or whether all involved alleles are suspect. Since most individuals are part of two HS families and are also often the parent of one or two HS families, alleles that are removed at one stage can be re-imputed in a next one.

The results of these decisions are that a missing haploblock allele is imputed, or an existing allele is rejected entirely, or an existing allele is updated by adding or removing marker data, or an allele (possibly missing) is left unchanged.

Conflicting scores may arise from different sources. The pedigree may be incorrect, a marker may be scored incorrectly in some individuals, the marker data may have been phased incorrectly, or a recombination may have occurred within a haploblock. Some pedigree errors will be easy to spot based on a limited number of markers and should be corrected before phasing and/or haploblock allele assignment. Remaining pedigree errors will likely result in many missing data either during the phasing of the marker data or in the haploblock allele assignment and may be identified in that way. Likewise, some unreliable markers will produce many conflicts with expected Mendelian inheritance patterns and can be removed before the haplotype allele assignment. Most remaining errors will be removed and where possible corrected during the allele assignment. Incorrectly phased marker data will be corrected in the same way, unless many individuals are involved; in that case, a large number of missing haploblock alleles will be assigned or the allele assignment process will not converge at all for that haploblock. Finally, a recombination event within a haploblock need not cause a serious problem if only affecting a founder or an individual without progeny. However, a recombination event in the middle of the pedigree will lead to incompatible alleles being assigned in earlier versus later generations, while probably the affected individual itself and some of its close relatives may be assigned missing haploblock alleles.

## Results

**Fig. 1:**
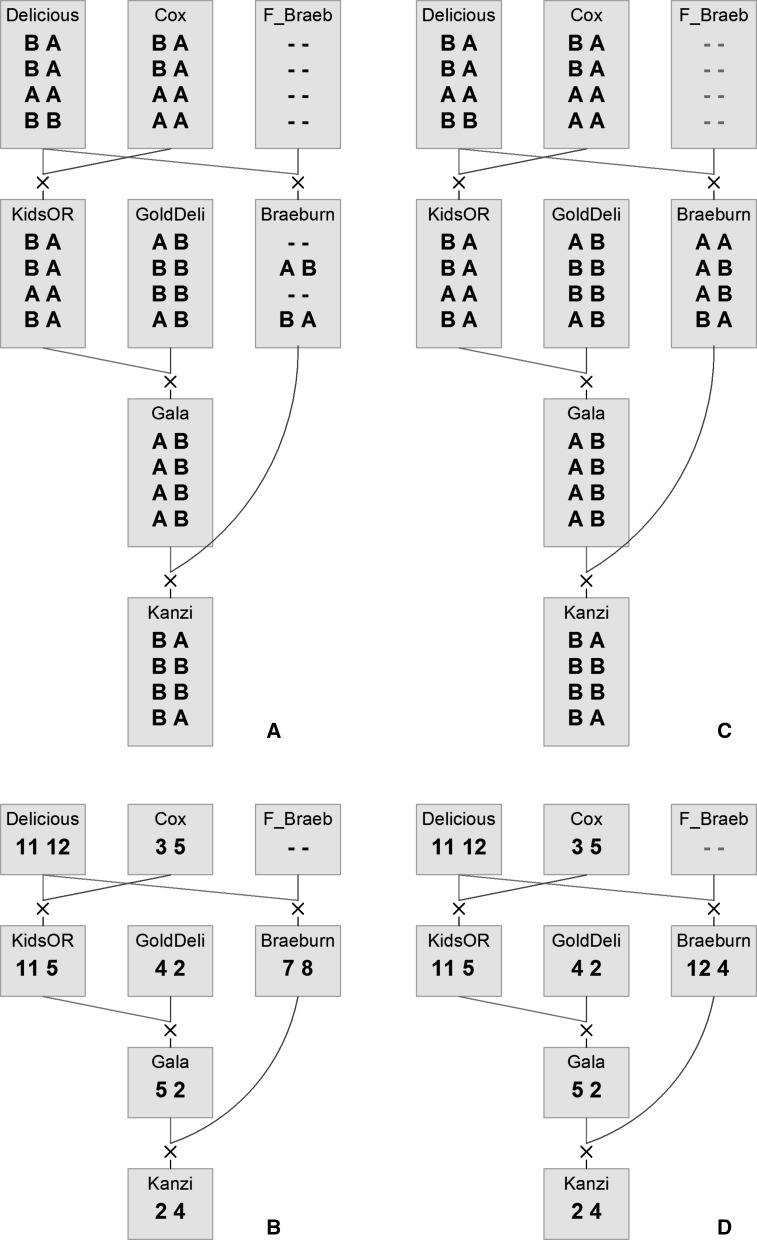
**a**, **c** Phased SNP alleles for 4 SNP loci in one haploblock; the allele *on the left* is inherited from the mother (*red link*), the one *on the right* from the father (*blue link*). **b**, **d** Haploblock alleles; each SNP allele configuration (including possible missing data) is assigned a unique allele number. **a**, **b** Original data. **c**, **d** after imputation. After imputation, the alleles are colour-coded: *black* are unchanged alleles, *red* are unchanged missing data, *magenta and cyan* are imputed alleles where original data were missing or different, respectively. (Color figure online)

A simple illustration of the allele assignment and marker imputation process is shown in Fig. 1. The figure is produced using the PediMap (Voorrips et al. 2012) software, for which the input files are prepared by PediHaplotyper. This figure illustrates imputation being performed (panel C vs. A, and D vs B), and also it shows how much more compact and more easy for the human investigator it is to follow a single haploblock allele (panels B and D) rather than multiple marker alleles (panels A and C).

**Fig. 2:**
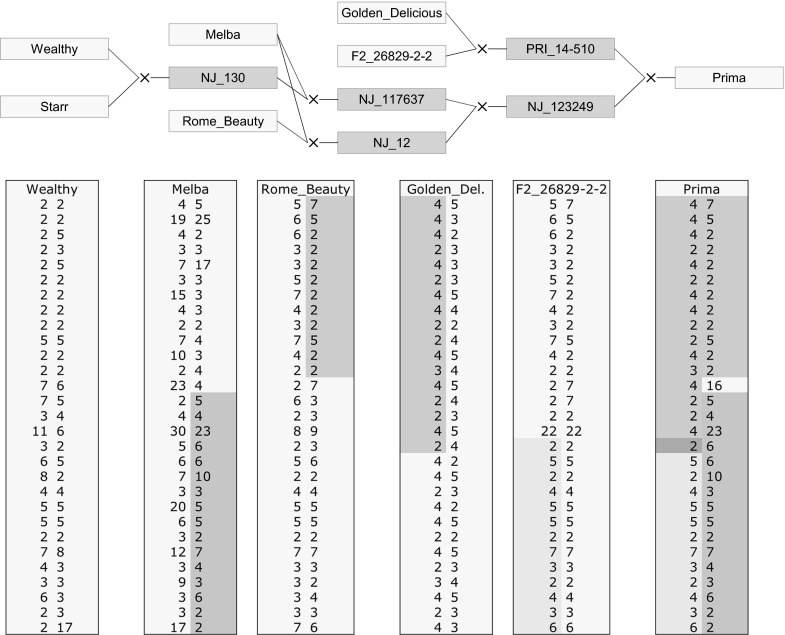
*Upper panel* Historic pedigree records of the apple cultivar Prima (Dayton et al. 1970; Evans et al. 2011); individuals shown in *red* are not available any more. Links to the female parents are shown in *red*, to the male parents in *blue*. *Lower panel* phased haploblock alleles for the first 36 cM of chromosome 11 of cv. Prima and five of its progenitors (the images for the full chromosome 11 are shown in Supplementary figure 1). The SNP genotypes of these six individuals could be phased, and haploblock alleles assigned, based on genotyping of additional, directly linked germplasm (not shown). Cv. Starr was genotyped but could not be phased due to lack of such directly linked genotyped germplasm. The (*orange, blue, green, and purple*) colouring highlights identical long-range haplotypes. Cv. Prima’s first haplotype originates from the top fragment from Golden Delicious haplotype 1 followed by a large segment from F2-26829-2-2. The site of recombination can be traced down to a window around one haploblock, as this haploblock is common to both grandparents. Prima’s second haplotype originates from the top segment of cv. Rome Beauty followed by a large segment from cv. Melba. The recombination must have occurred within the 13th haploblock, which explains the conflicting allele of this haploblock in Prima’s paternal haplotype. (Color figure online)

Haploblock allele assignment might be expected to produce inconsistent results in disconnected parts of the pedigree, such as in progenitors and progeny of some intermediate generations of non-genotyped individuals. However, in practice, such inconsistent results do not seem to happen often; an example taken from a larger apple pedigree is shown in Fig. 2 and Supplementary figure 1. The consistency in haplotype data between cv. Prima and its ancestors in the absence of data for 1–2 intermediate generations shows the power of the current approach.

The advantages of haploblocks over SNP markers for Bayesian QTL mapping were compared by analysing a test data set from an apple pedigree of 744 individuals using FlexQTL software (Bink et al. 2014; publicly available from www.flexqtl.nl). A total of 7549 high-quality SNP markers were available in this pedigree (Van de Weg, unpublished) from the 20 K Infinium^®^ SNP array (Bianco et al. 2014). In a pre-processing step, the SNP markers were phased with FlexQTL software and grouped into 1112 haploblocks of varying sizes; haploblock alleles were assigned by PediHaplotyper. On average, about 27 % of the meioses were informative per SNP marker and about 78 % per haploblock. The QTL analysis with all 7549 SNPs was aborted after estimating that it would take at least 1700 h (while requiring 2.3 GB of memory), the analysis with a subset of 1276 SNPs (every 6th SNP and the first and last SNP of a linkage group) finished in 59:07 h (0.17 GB), and the one with 1112 haploblocks took 49:00 h (0.14 GB). For this test data set, the QTL analyses with 1276 SNPs and with 1112 haploblocks yielded very similar QTL inferences (Appendix), indicating that marker density was not the limiting factor for QTL discovery. Use of the full SNP data set, either as single SNPs or haploblocks, is nevertheless preferable for use in downstream analyses as it may result in more accurate estimates of IBD-probabilities (Supplementary figure 2) and a better selection of predictive markers for use in marker-assisted breeding (MAB).

The pedigree of this example was optimized for phasing of SNP data: successive generations of un-genotyped progenitors were removed (e.g. the pedigree of Prima, Fig. 2). When the deleted individuals and pedigree relations were restored for intermediate generations (with missing data for the markers or haploblocks) and the pedigree was re-analysed, phasing errors occurred more frequently with the SNP data than with the corresponding haploblock data, showing the use of haploblocks to fix and maintain earlier phasing results.

## Application

**Fig. 3:**
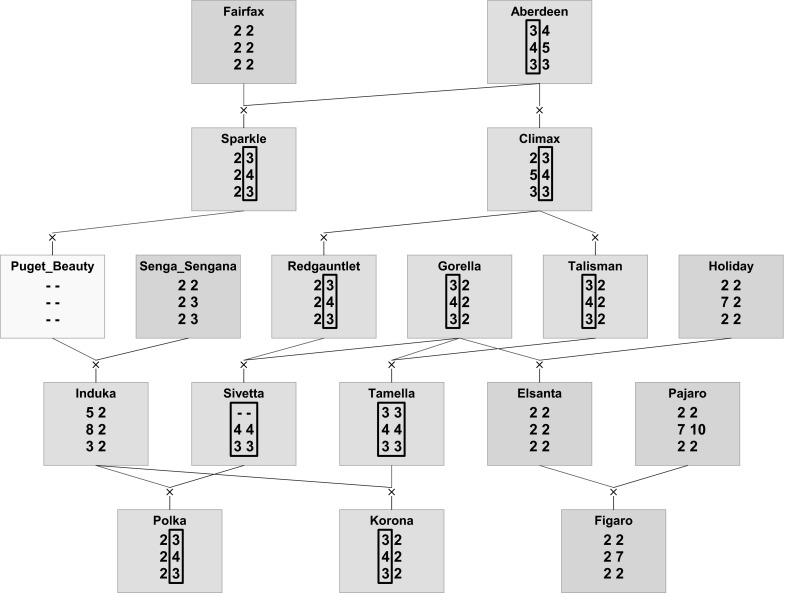
Flow of a chromosome segment consisting of three haploblocks along a pedigree in strawberry. *Green *(*blue*) boxes indicate individuals that show (no) disease resistance, the yellow individual was not phenotyped and genotyped. The resistance is associated with the haplotype consisting of haploblock alleles 3-4-3; the resistance in “Induka” may be due to another resistance gene from Puget Beauty (not shown). (Color figure online)

PediHaplotyper was used in two other multi-family QTL discovery studies for bud break and flowering time in apple (Allard et al. 2016) and fruit quality traits in peach (Hernández Mora et al. unpublished). PediHaplotyper has also been applied in the allo-octoploid strawberry to trace inheritance patterns in a pedigree using SNP data from a 90 K Axiom array (Bassil et al. 2015) (Fig. 3).

## Discussion

The representation of phased marker genotypes as multi-marker haploblock alleles makes it more easy for the human investigator to understand the inheritance of chromosome segments through a pedigree. This in turn helps to identify and correct errors in assumed pedigree relations and in phased marker genotypes and is therefore very useful in producing a high-quality data set that can be used for genetic studies, including QTL mapping. The PediHaplotyper software we present here takes as input phased marker genotypes and pre-defined haploblocks. These have to be generated using other software; in our case, we used FlexQTL (Bink et al. 2014).

Marker data need to be generally consistent, although occasional inconsistencies are handled well by PediHaplotyper. A common cause of inconsistent SNP marker genotypes is the occurrence of null alleles (Pikunova et al. 2014); null allele heterozygotes are commonly assigned the homozygous genotype of the observed allele by SNP genotyping software. The ASSIsT software (Di Guardo et al. 2015) addresses this issue, currently only for Infinium SNP arrays, and FlexQTL™ supports their discovery through its report on (in)consistencies in marker data between successive generations.

PediHaplotyper assumes that no recombination occurs within a haploblock over the entire pedigree. If a recombined haploblock allele appears in an individual without progeny that does not have serious consequences, most likely the individual will be assigned a missing haploblock allele. A recombination within a haploblock occurring earlier in the pedigree can be spotted due to incompatible alleles being present in generations before and after the recombination, allowing to redefine the haploblocks involved.

In some cases, the haploblock alleles assigned by PediHaplotyper may appear to be inconsistent even if the underlying marker data are consistent. This apparent inconsistency is due to markers within a haploblock with missing genotypes in part of the pedigree and insufficient linking information to update their genotypes from other parts of the pedigree where they are scored. In that case, haploblock alleles are assigned that differ only in missing marker data. As these alleles have different IDs (numbers), they will be considered as different by e.g. QTL mapping software. Output files with the marker haplotypes of all haploblock alleles are provided that allow to resolve this issue. Moreover, for easy visualization, PediHaplotyper can optionally include the number of markers with missing values in the names of haploblock alleles.

Haploblock alleles can also help to identify unknown pedigree relations. For example in Fig. 2, there are two non-genotyped generations between cv Prima and its great-grandparent cv Melba. Because of the problems in inferring linkage phases across multiple generations of missing data, Prima was entered as a founder with unknown parents in the phasing and haploblock assignment steps. The haploblock genotypes assigned to Melba and Prima are therefore not affected by their direct pedigree link. If the relation between Melba and Prima were unknown, the large corresponding segment of chromosome 11 would clearly indicate that Melba could be a progenitor of Prima or else a very close relative. With further data from the other 16 chromosomes, the evidence for Melba rather than other candidates being an ancestor of Prima would further increase.

Haploblocks are more efficient than SNP markers in QTL mapping. As the information present in individual SNP data is represented in a more compact form in haploblocks, the computational requirements (memory and processor time) for QTL mapping are (very) much lower with haploblocks. In QTL discovery, results are not much affected by the use of haploblocks versus SNPs: while the number of informative meioses per haploblock is higher than that per SNP, the total number of available meioses, and therefore also the power remains the same. As illustrated in the QTL analysis example, severely reducing the number of SNPs did not cause a significant change in the resulting QTL model but led to incorrect IBD estimates in some individuals; it also decreases the possibility to select optimal predictive markers for MAB.

In conclusion, haploblock genotypes as assigned by PediHaplotyper can be of great value for resolving issues with pedigree structure, marker data and marker phasing, and also increase the computational efficiency of QTL mapping in large pedigrees, allowing to use data sets with more markers, thus increasing overall reliability of IBD estimates.

## Electronic supplementary material

Below is the link to the electronic supplementary material.

Supplementary material 1 (PDF 434 kb)

## Appendix: QTL fitting with FlexQTL based on single SNPs or haploblocks

### Model settings

- Prior and max number of QTL set at 10 and 20, respectively.
- Additive QTL model with normal prior distribution.

### MCMC settings

- 100 K iterations, using a thinning of 100,
- Number of marker loci that are jointly updated with respect to linkage phase among marker (and QTL) alleles: 5.
- Sampling of marker information was performed every 1000th iteration.

### QTL inferences

- Convergence as evaluated by effective chain sizes for four parameters:

**Table:**

	SNP	HB
Mean	126	399
Variance error	477	222
nQTL	546	403
varQTL	248	341

- Number of QTL with a Bayes Factor >5: three for both marker types; Bayes factors for these three QTL:

**Table:**

	SNP	HB
QTL-1	28	28
QTL-2	12	25
QTL-3	6	5

- QTL were at the same positions for SNPs and haploblocks (not shown).
