# Supplementary material for: PediHaplotyper: software for consistent assignment of marker haplotypes in pedigrees
Source: Mol Breed. 2016 Aug 8;36:119. doi: 10.1007/s11032-016-0539-y (PMC4977329; doi:10.1007/s11032-016-0539-y)
Supplement: Supplementary file 1 — Supplementary material 1 (PDF 434 kb) [file 11032_2016_539_MOESM1_ESM.pdf]

Wealthy		Melba		Rome_Beauty		Golden_Del.		F2_26829-2-2		Prima	
2	2	4	5	5	7	4	5	5	7	4	7
2	2	19	25	6	5	4	3	6	5	4	5
2	5	4	2	6	2	4	2	6	2	4	2
2	3	3	3	3	2	2	3	3	2	2	2
2	5	7	17	3	2	4	3	3	2	4	2
2	2	3	3	5	2	2	3	5	2	2	2
2	2	15	3	7	2	4	5	7	2	4	2
2	2	4	3	4	2	4	4	4	2	4	2
2	2	2	2	3	2	2	2	3	2	2	2
5	5	7	4	7	5	2	4	7	5	2	5
2	2	10	3	4	2	4	5	4	2	4	2
2	2	2	4	2	2	3	4	2	2	3	2
7	6	23	4	2	7	4	5	2	7	4	16
7	5	2	5	6	3	2	4	2	7	2	5
3	4	4	4	2	3	2	3	2	2	2	4
11	6	30	23	8	9	4	5	22	22	4	23
3	2	5	6	2	3	2	4	2	2	2	6
6	5	6	6	5	6	4	2	5	5	5	6
8	2	7	10	2	2	4	5	2	2	2	10
4	4	3	3	4	4	2	3	4	4	4	3
5	5	20	5	5	5	4	2	5	5	5	5
5	5	6	5	5	5	4	5	5	5	5	5
2	2	3	2	2	2	2	2	2	2	2	2
7	8	12	7	7	7	4	5	7	7	7	7
4	3	3	4	3	3	2	3	3	3	3	4
3	3	9	3	3	2	3	4	2	2	2	3
6	3	3	6	3	4	4	5	4	4	4	6
2	3	3	2	3	3	2	3	3	3	3	2
2	17	17	2	7	6	4	3	6	6	6	2
3	2	2	2	2	3	3	2	3	3	3	2
6	5	5	4	5	2	4	5	2	2	2	4
6	4	25	4	3	5	4	4	5	5	5	4
3	2	2	2	2	2	2	2	2	2	2	2
4	14	21	14	6	4	4	4	4	4	4	14
4	3	15	3	4	4	4	2	4	4	4	3
3	2	3	2	3	3	3	2	3	3	3	2
4	11	20	11	4	4	4	2	4	4	4	11
3	2	3	2	3	3	3	2	3	3	3	2
3	3	3	3	3	3	4	2	3	3	3	3
6	18	6	18	3	6	4	5	6	6	6	18
3	16	24	16	4	3	4	5	3	4	3	16
6	4	4	4	4	6	4	5	6	4	6	4
7	13	4	13	4	5	4	5	5	4	5	13
7	2	4	2	4	5	4	5	5	4	5	2
8	11	6	11	7	7	4	5	7	7	7	11
2	2	3	2	3	3	2	2	3	3	3	2
5	6	11	6	3	3	3	4	3	3	3	6
9	17	8	17	7	7	4	5	7	7	7	17
8	15	7	15	5	5	4	5	16	5	5	15
3	4	4	4	3	3	2	3	3	3	3	4
6	3	5	3	5	5	4	5	5	5	5	3
7	3	6	3	4	4	2	4	4	4	4	3
5	13	3	13	3	3	4	3	3	3	3	13
8	14	7	14	6	6	4	5	6	6	6	14
4	3	3	3	3	3	4	4	3	3	3	3
6	3	12	3	5	5	2	4	5	5	5	3
7	6	5	6	6	6	4	5	6	6	6	6
4	4	4	4	3	3	4	4	3	3	3	4
8	9	19	9	6	6	4	5	6	6	6	9
2	4	4	4	4	4	2	4	4	4	4	4
4	2	2	2	5	5	4	2	5	5	5	2
2	4	3	4	2	3	4	2	3	2	3	4
4	2	2	2	2	6	4	2	8	2	8	2
2	2	4	2	2	2	2	2	2	2	2	2
2	3	2	3	2	2	2	2	2	2	2	3
7	14	18	14	2	7	4	4	2	2	2	14
2	2	2	2	2	2	2	2	2	2	2	2
7	4	6	4	8	7	4	5	8	8	8	4
5	11	6	11	7	5	4	5	7	7	7	11
8	5	19	5	3	7	4	5	3	3	3	5
8	4	28	4	6	7	4	5	6	6	6	4
5	4	4	4	3	6	4	2	3	3	3	4

Supplementary figure 1. The alleles for all 72 haploblocks of chromosome 11 (covering 83 cM), for the six apple genotypes shown in Figure 2B. This illustrates the fidelity of the haplotyping and haploblock allele assignment process: except for the recombination point there is only one inconsistency among the 72 haploblocks.

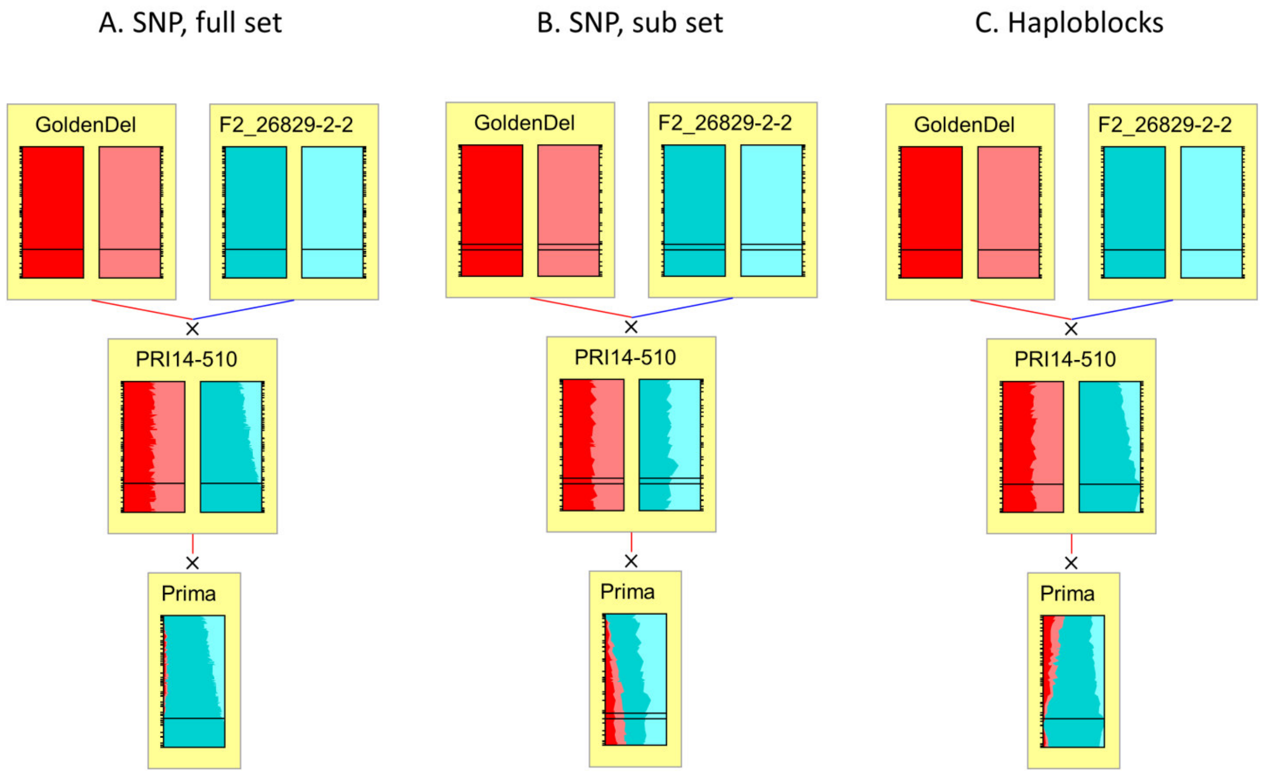

Supplementary figure 2. IBD estimates for the maternal homolog of chromosome 1 of the apple cultivar 'Prima' for three different marker sets: A the full set of 286 SNP (**A**), a subset of 49 SNP (Every 6th SNP of the full set and the first and last marker of the chromosome) (**B**), and a set of 47 haploblocks representing the full set of SNP. The position of a marker for the *Vf* gene for resistance to *Venturia inaequalis* is marked by a horizontal line in panels A & C, and by its flanking markers in panel B. The allele for Vf-resistance, which originated from "*Malus floribunda* 821" was introgressed into current modern germplasm through the breeding selection F2-26829-2-2, and is known to be present in Prima (Dayton et al. 1970). In the non-genotyped individual "PRI 14-510" and the genotype "Prima" this genetic relationship was traced with 100% probability with the full SNP and the haploblock dataset, but remained unclear with the SNP subset due to absence of founder specific markers. (The figure was prepared in Pedimap (Voorrips et al 2012) and shows for each individual the IBD probabilities of the maternally and paternally inherited gametes. The vertical dimension represents the genetic map of linkage group; the width of each colour at a certain height represents the IBD probability of the founder alleles at that genetic position.)
